# Case Report: Compound heterozygous *CEP152* c.3346-5T>C variant and chr15 deletion causing recurrent MCPH–SCKS in a Chinese pregnant woman across two consecutive pregnancies

**DOI:** 10.3389/fgene.2025.1646297

**Published:** 2025-11-12

**Authors:** Tao Zhang, Hua Yuan, Xiaoliang Shi, Yao He, Haitao Pan, Yongxing Zhong, Jintang Zhang, Zhen Yang, Yunyan Ke, Yan Chen, Feng Zhang

**Affiliations:** 1 Shaoxing Maternity and Child Health Care Hospital, Shaoxing, Zhejiang, China; 2 Obstetrics and Gynecology Hospital of Shaoxing University, Shaoxing, Zhejiang, China; 3 Clinic Lab, BGI Genomics, Shanghai, China; 4 Shaoxing Hospital of Traditional Chinese Medicine, Shaoxing, Zhejiang, China

**Keywords:** primary autosomal recessive microcephaly, seckel syndrome, *CEP152* gene, whole-exome sequencing, prenatal diagnosis

## Abstract

**Background:**

Primary autosomal recessive microcephaly and Seckel syndrome spectrum (MCPH–SCKS) disorders are a group of autosomal recessive conditions characterized by severe growth retardation and neurodevelopmental impairment. *CEP152* variants are established causes of both microcephaly and Seckel syndrome phenotypes, but the pathogenicity of different variant combinations and their clinical recurrence patterns require further verification with additional cases.

**Methods:**

Clinical and genetic analyses were performed for two consecutive pregnancies of a non-consanguineous Chinese couple. Fetal phenotypes were evaluated by ultrasound and fetal MRI sequentially; copy number variation sequencing (CNV-seq) was used to detect large genomic variants, whole-exome sequencing (WES) to screen for point variants, and minigene splicing assays to characterize the functional impact of key variants.

**Results:**

Fetuses in both pregnancies were diagnosed with MCPH–SCKS and harbored identical compound heterozygous CEP152 variants: a paternally inherited splice-site variant c.3346–5T>C and a maternally inherited 129.6 kb chromosomal deletion localized to 15q21.1, encompassing the entire *CEP152* gene. Minigene assays confirmed that the c.3346–5T>C variant caused aberrant splicing via intron retention and exon skipping, clarifying its pathogenicity. The second pregnancy in 2024 independently verified the pathogenicity of this variant combination and disease recurrence.

**Conclusion:**

This study represents the first report of identical compound heterozygous *CEP152* variants causing MCPH–SCKS in two consecutive pregnancies. The independent occurrence in the second pregnancy not only confirms the pathogenicity of this variant combination but also provides clinical evidence for the 25% recurrence risk of autosomal recessive disorders. Furthermore, this report underscores the critical importance of comprehensive genetic testing, including CNV analysis, for the prenatal diagnosis of MCPH–SCKS, offering valuable guidance for genetic counseling and prenatal intervention in similar families.

## Introduction

Primary autosomal recessive microcephaly and Seckel syndrome spectrum (MCPH–SCKS) disorders contain a heterogeneous group of autosomal recessive inherited diseases, which are characterized by primary (congenital) microcephaly, visceral abnormalities, and a variable degree of cognitive impairment, facial dysmorphism, and short stature. To date, 28 MCPH loci (MCPH1–MCPH28), including *CEP152*, have been described worldwide, encompassing the following genes: MCPH1 (*BRIT1*), MCPH2 (*WDR62*), MCPH3 (*CDK5RAP2*), MCPH4 (*CASC5*), MCPH5 (*ASPM*), MCPH6 (*CENPJ*), MCPH7 (*STIL*), MCPH8 (*CEP135*), MCPH9 (*CEP152*), MCPH10 (*ZNF335*), MCPH11 (*PHC1*), MCPH12 (*CDK6*), MCPH13 (*CENPE*), MCPH14 (*SAS6*), MCPH15 (*MFSD2A*), MCPH16 (*ANKLE2*), MCPH17 (*CIT*), MCPH18 (*WDFY3*), MCPH19 (*COPB2*), MCPH20 (*KIF14*), MCPH21 (*NACAPD2*), MCPH22 (*NACAPD3*), MCPH23 (*NCAPH/BRRN*), MCPH24 (*NUP37*), MCPH25 (*MAP11*), MCPH26 (*LMNB1*), MCPH27 (*LMNB2*), and MCPH28 (*RRP7A*) (Author anonymous, 2020; Jayaraman et al., 2018; Farooq et al., 2020). These 28 MCPH loci have clarified our understanding of the molecular basis of the microcephaly genetic disorder.

The protein-coding gene *CEP152* is located on chromosome 15q21.1, spans 149368 bp, includes 38 exons, and encodes 1710 amino acids with a molecular mass of 195,626 Da (Jamieson et al., 2000). It is also known as Asterless, *KIAA0912*, *SCKL5*, and *MCPH9* (Dzhindzhev et al., 2010). In cells, CEP152 is located in the centrosome and encodes a protein that is considered to be relevant to centrosome function. In addition, centrosome protein CEP152 is a novel protein involved in apoptosis (Nyberg et al., 2002). Variants in *CEP152* are a rare cause of MCPH but a frequent cause of Seckel syndrome (Barbelanne and Tsang, 2014; Andersen et al., 2003; Guernsey et al., 2010).

Although compound heterozygous *CEP152* variants have been reported in MCPH–SCKS cases, critical gaps remain in the current literature. For instance, Hong L. et al. (2019) described a Chinese patient with MCPH–SCKS harboring compound heterozygous *CEP152* NM_001194998.1 variants: a missense variant c.1535T>A (p.Tyr512Asn) and a splice-site variant c.3346–5T>C. However, the c.3346–5T>C variant in this study was classified as a variant of unknown significance (VUS) in the ClinVar database (accession: VCV003382300.1) due to the lack of functional validation. To date, no studies have reported the combination of *CEP152* NM_001194998.1 c.3346–5T>C with a large genomic deletion, encompassing the entire *CEP152* locus as the genetic cause of MCPH–SCKS. Moreover, recurrent MCPH–SCKS caused by any *CEP152* compound heterozygous variants in two consecutive pregnancies has not been described in the literature. These gaps limit our understanding of *CEP152* variant spectra and the recurrence risk of MCPH–SCKS, highlighting the need for additional clinical and functional evidence.

In this study, we reported a Chinese fetus patient who presented fetal microcephaly on ultrasound screening and MRI, and subsequent genetic testing identified the related gene mutation. Copy number variation sequencing (CNV-seq) revealed that the proband had a heterozygous deletion of approximately 129.6 kb in the chr15:49030446–49160066 region, which contained the *CEP152* gene. Then, whole-exome sequencing (WES) revealed a variant, c.3346–5T>C, at chr15:49044671, which is relevant to two diseases: primary microcephaly type and Seckel syndrome. Sanger sequencing verification confirmed that the proband harbored compound heterozygous pathogenic variants in *CEP152*: the 129.6 kb deletion was inherited from the mother, a heterozygous carrier of this deletion, and the c.3346–5T>C variant was inherited from the father, a heterozygous carrier of this variant.

## Materials and methods

### Sample collection

The study was approved by the Institutional Ethics Committee of Shaoxing Maternity and Child Healthcare Hospital. The family members had signed informed consent documents. Parental consent was obtained for collecting the prenatal fetal amniotic cell samples at 27+5 weeks of pregnancy. Peripheral blood samples were also collected from the proband’s parents.

### Copy number variation sequencing and whole-exome sequencing

Genomic DNA was extracted from the proband and parental blood using DNeasy Blood and Tissue Kits (QIAGEN, Hilden, Germany), according to the manufacturer’s procedures.

For WES, DNA libraries were constructed, which were subjected to whole-exome capture on an Agilent SureSelect Low Input Reagent Kit (Agilent, California, United States). We then performed high-throughput sequencing on the Illumina HiSeq X Ten platform (Illumina, Inc., San Diego, CA, United States) (150-bp paired-end reads) to analyze point variants and small deletions or insertions. Its bioinformatic pipeline included FastQC (v0.11.9) for filtering low-quality reads (Phred <20) and adapters, alignment to the GRCh38/hg38 reference genome via BWA-MEM (v0.7.17); Picard Tools (v2.27.5) for duplicate removal and sorting; and GATK (v4.3.0.0) for base recalibration. GATK HaplotypeCaller was used for SNV/Indel calling (joint genotyping for family samples), followed by filtering (DP ≥ 10, AB ≥ 0.2 for heterozygotes, MAF <0.01 in gnomAD/ExAC, and ≥2 tools predicting pathogenicity). The quality control of sequencing data showed a total of 20,858 genes, 34962037 bp of target area length, 99.87% of target area coverage, 111.277 × of average sequencing depth in the target region, and 99.09% of proportion in the target area with an average depth of >20 ×, suggesting that the results of WES sequencing were reliable.

For CNV-seq, genomic DNA was fragmented using Hieff NGS® Fast-Pace^TM^ DNA Fragmentation Reagent and prepared for the PCR-free library using the Hieff NGS® Complete Adapter Kit for Illumina®. DNA was fragmented using Hieff NGS® Fast-Pace^TM^ Reagent (Yeasen), and libraries were built with the Hieff NGS® Complete Adapter Kit (Yeasen), then sequenced on the Illumina NovaSeq 6000 Platform (150-bp paired-end reads). Its pipeline included FastQC filtering (same as WES), alignment to GRCh38/hg38 via BWA-MEM, Picard Tools for duplicate removal; SAMtools (v1.15.1) for 100-bp bin read counting, CNVkit (v0.9.10) for GC/depth normalization and CNV calling (minimum size 10 kb), and ANNOVAR (v20210802) for annotation against DGV/ClinVar. CNV-seq quality control showed an average genome depth of 30 ×, >98% mapping rate, and GC content consistent with the reference genome.

### Variant evaluation

The QC (PMID: 28361673) was used to evaluate the sequencing quality of the original data, and the low-quality and contaminated reads were removed. The variant was evaluated by dbSNP (http://www.ncbi.nlm.nih. gov/projects/SNP/), 1,000 Genomes (http://www.1000genomes. org/), ExAC (http://exac.broadinstitute.org/), gnomAD (http://gnomad.broadinstitute.org/), ESP6500 (http://evs.gs.washington. edu/EVS/), the Human Gene Mutation Database (HGMD; http://portal.biobase-international.com/hgmd/pro/start.php), and Mutation Taster (http://mutationtaster.org). The dbNSFP database was used to predict the pathogenicity of missense mutations and splice mutations. Novel variants were recognized using the HGMD and ClinVar (PMID:29165669; PMID:32596782). Sanger sequencing was used to validate the candidate variants in all family members. All mutation sites were classified according to ACMG genetic variation classification criteria and guidelines.

### Bacterial strains, plasmids, primers, and minigene vector construction

For this study, we employed *E. coli* DH5α for all cloning procedures. Using the pcMINI and pcMINI-C vectors as backbones, we constructed both wild-type and mutant fragments containing *CEP152* exons and introns. Initial target fragment amplification was performed through nested PCR from normal human genomic DNA using primer pairs 52228-F/54461-R and 52436-F/54180-R. The 1,284 bp pcMINI-*CEP152* wild-type fragment was directly amplified with pcMINI-*CEP152*-KpnI-F/XhoI-R primers, while the mutant version was generated via overlap PCR by separately amplifying left (545 bp) and right (770 bp) halves before combining them at a 1:1 ratio for full-length amplification. Similarly, the 1811 bp pcMINI-C-*CEP152* wild-type was obtained by amplifying left (1,200 bp) and right (641 bp) segments, followed by 1:1 mixing, whereas the mutant required three-segment amplification (513 bp, 718 bp, and 641 bp) combined at a 1:1:1 ratio before final amplification. All primer sequences are provided in Supplementary Table S1.

### Minigene splicing assay

The recombinant vectors were transiently transfected into HeLa and 293T cell lines, and the transfection steps were carried out according to the instructions of the liposome manufacturer. Total RNA was extracted from the cell samples after 48 h, and the extraction method was performed according to the kit instructions. After determining the concentration, cDNA was synthesized by reverse transcription using an equal volume of RNA. pcMINI-wt/mut was amplified by PCR with the primers pcMINI-F/pcMINI-R; pcMINI-C-wt/mut was amplified by PCR with the primers pcMINI-C-F/pcMINI-C-R, and the gene transcripts were detected by agarose gel electrophoresis. Each band was retrieved separately for Sanger sequencing.

## Results

### Case presentation

**FIGURE 1:**
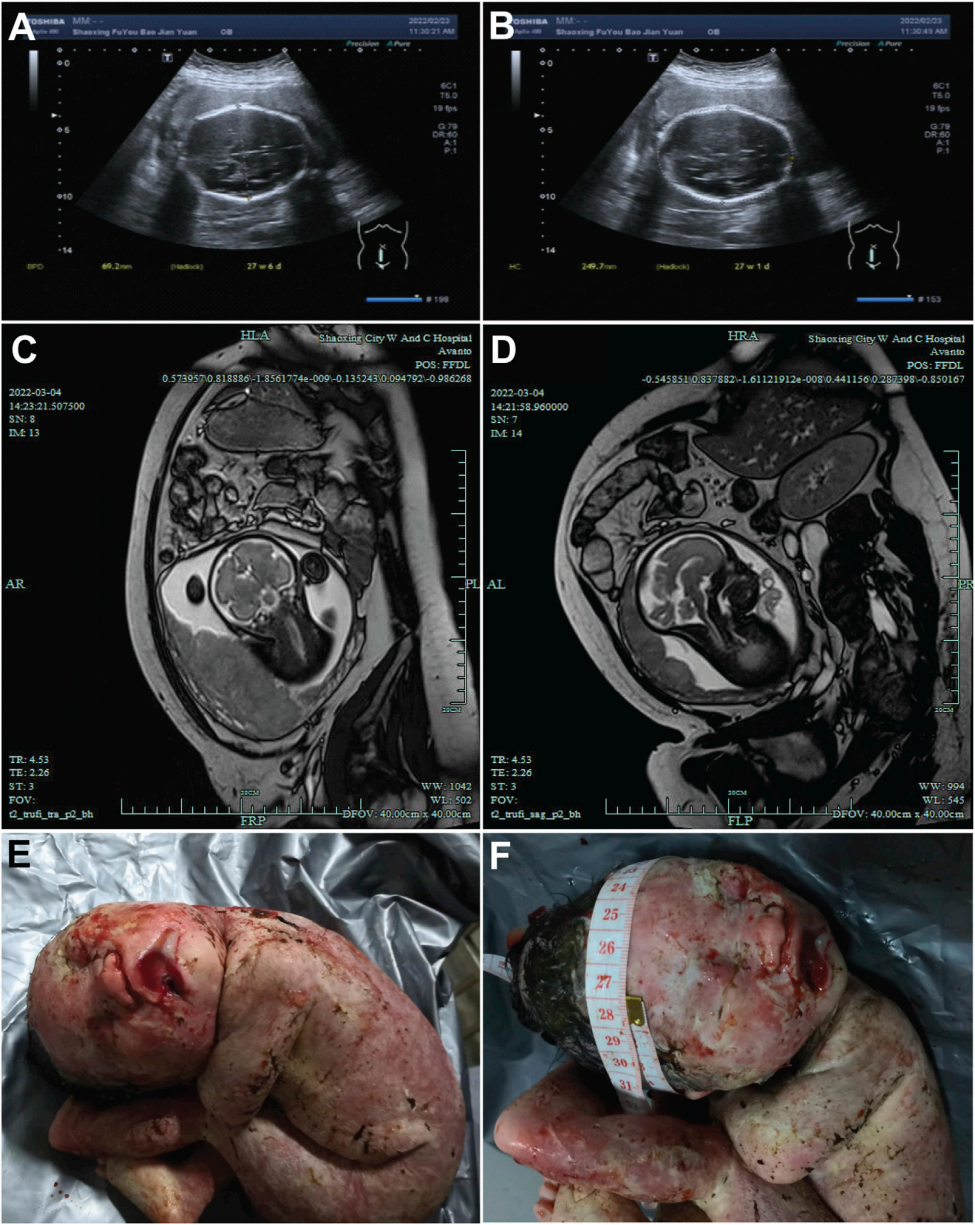
Ultrasound and magnetic resonance images of the fetal head. **(A,B)** Ultrasound images of the fetal head at 27 weeks + 4 days of gestation, showing the fetal head from different sections. The measured values indicated in the figures (such as 46.2 mm and 240.7 mm) represent the relevant diameters of the fetal head in the corresponding sections. **(C,D)** MRI images of the fetal head at 27 weeks +4 days of gestation, presenting the structure of the fetal head from different angles. **(E,F)** Post-induction labor photographs of the fetus. E presents the general appearance of the fetus. F focuses on the fetal head, where a measuring tape is used to document the head circumference.

The proband’s mother was a 30-year-old Chinese Han woman with regular 30-day menstrual cycles and no significant medical history. Her pregnancy was confirmed after 30 days of amenorrhea with a positive urine test. She received standard prenatal care, including 0.4 mg/day folic acid supplementation during the first trimester, with normal oral glucose tolerance test results and no exposure to teratogens. Initial prenatal screening at 13+1 weeks showed a normal nuchal translucency measurement (2.8 mm) and low-risk non-invasive prenatal testing results. However, subsequent ultrasounds revealed progressively severe fetal growth abnormalities, with head circumference measurements declining to the 0.1st percentile by 24+2 weeks and estimated fetal weight below the 1st percentile (Figures 1A,B). Fetal MRI at 30+6 weeks confirmed significant microcephaly (head circumference 266 mm, equivalent to 28 weeks’ gestation) (Figures 1C,D). Following a multidisciplinary evaluation at 31+5 weeks that confirmed severe fetal growth restriction with microcephaly, the parents elected to terminate the pregnancy after ethical committee approval. The male fetus was delivered stillborn without apparent dysmorphic features. Postmortem genetic testing was performed to determine the etiology of these findings, with the aim of guiding future pregnancies. The parents had no family history of genetic disorders or previous adverse pregnancy outcomes.

### Whole-exome sequencing and mitochondrial genome findings in the proband

**FIGURE 2:**
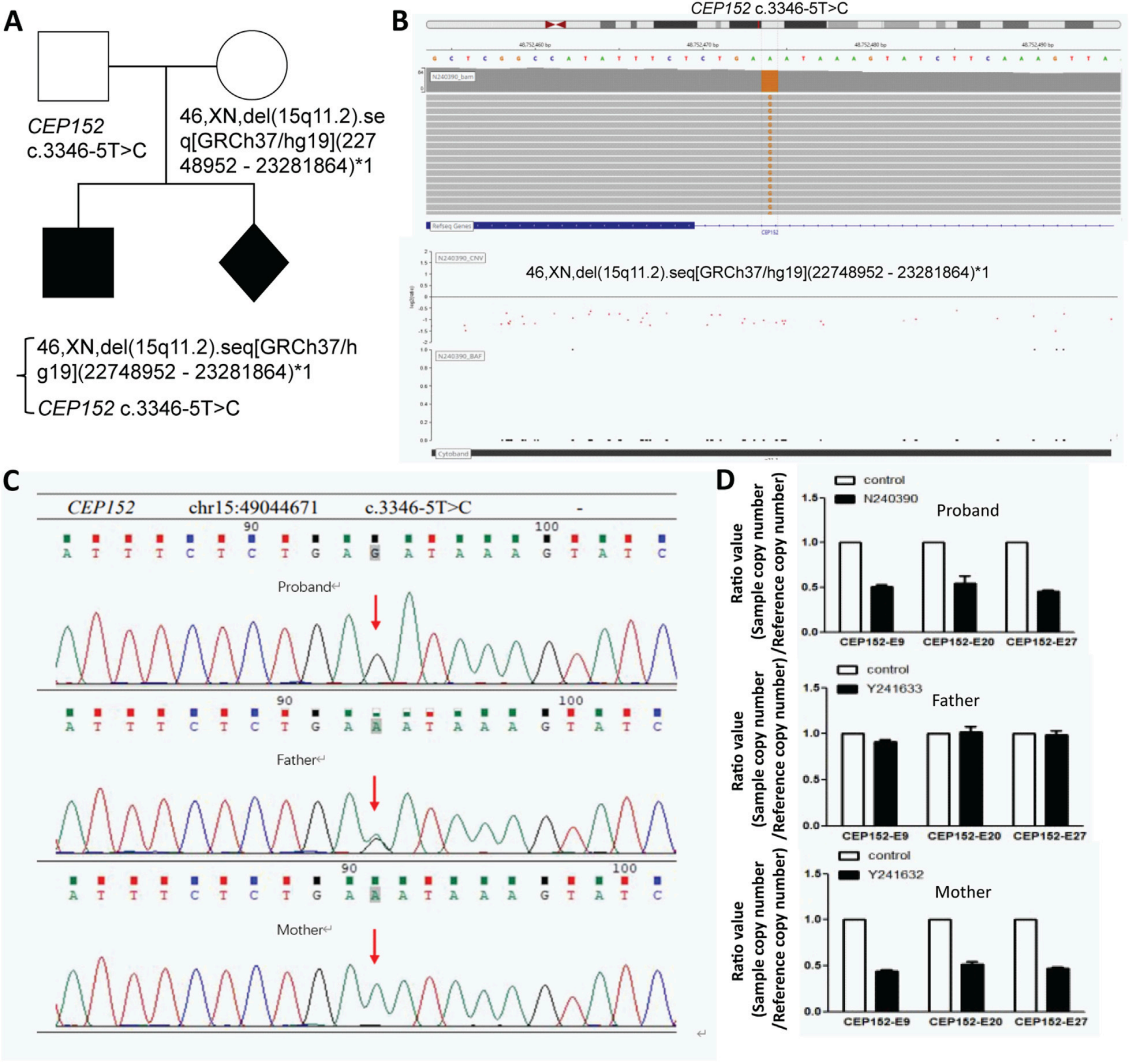
Genetic analysis of *CEP152* variants and deletions in this family with fetal abnormality. **(A)** Pedigree of the family. **(B)** Visualization of the *CEP152* c.3346–5T>C variant (top) and the 46,XN,del (15q11.2). seq [GRCh37/hg19](22748952–23281864)*1 deletion (bottom) using bioinformatics tools. **(C)** Sanger sequencing results for the *CEP152* gene. The red arrow points to the position of the **(C)**3346–5T>C variant. **(D)** Copy number variation (CNV) analysis results. The ratio values (sample copy number/reference copy number) for different exons (*CEP152*-E9, *CEP152*-E20, and *CEP152*-E27) are presented for the proband, father, and mother.

We performed whole-exome sequencing (WES) with blood total genomic DNA extracted from the proband and his parents. Furthermore, from WES and mitochondrial genome detection, two conclusions can be derived. First, no pathogenic or suspected pathogenic variants consistent with the patient’s phenotype have been detected; second, the pathogenic evidence is insufficient, but it is consistent with the patient’s phenotype, and two possible variants of pathogenesis are not excluded: one is the *VPS13B* gene with an variant of c.5692G>A, p. Glu1898lys (nucleotide of coding region 5,692 changed from guanine to adenine, resulting in amino acid 1898 changed from glutamic acid to lysine); this is a missense variant, and the disease correlated is Cohen syndrome. The other is the *CEP152* gene at chr15:49044671 with a variant of NM_001194998.1 c.3346–5T>C relevant to two diseases—primary microcephaly type and Seckel syndrome (Figures 2A,B). Both variants were validated by Sanger sequencing to confirm their authenticity (Figure 2C).

### CNV analysis of the proband and family

Through CNV analysis, it was found that the subjects had a heterozygous deletion of approximately 129.6 kb in the chr15:49030446–49160066 region, which contained the *CEP152* and *SHC4* genes. The absence of this area was not recorded in the population database (DGV). There is no record in the ClinGen database. No record in the ClinVar database. None in the disease database deciphered the record. The *CEP152* gene is associated with primary autosomal recessive microcephaly (MCPH) and Seckel syndrome (SCKS). After verification of the CNV results of the proband and the proband’s father and mother, it is found that the variant type of the proband was hemizygous, and the father harbored a heterozygous variant, while the mother was a carrier of the deletion in the relevant fragment of the *CEP152* gene (Figure 2D; Supplementary Figure S1). According to the American College of Medical Genetics and Genomics (ACMG) guidelines, the NM_001194998.1 c.3346–5T>C variant was predicted to be pathogenic. In 2024, the patient achieved a spontaneous pregnancy. Prenatal diagnosis confirmed recurrence of the same genetic syndrome, with the fetus harboring compound heterozygous *CEP152 NM_001194998.1* variants: a paternally inherited hemizygous c.3346–5T>C splice-site variant and a maternally inherited heterozygous deletion represented as 46,XN,del (15q11.2). seq [GRCh37/hg19](22748952–23281864)*1, which was approximately 129.6 kb and spanned the entire *CEP152* locus. After multidisciplinary consultation at a tertiary genetics center, the family elected to terminate the pregnancy.

### Splicing assays of *CEP152* and transcript analysis

**FIGURE 3:**
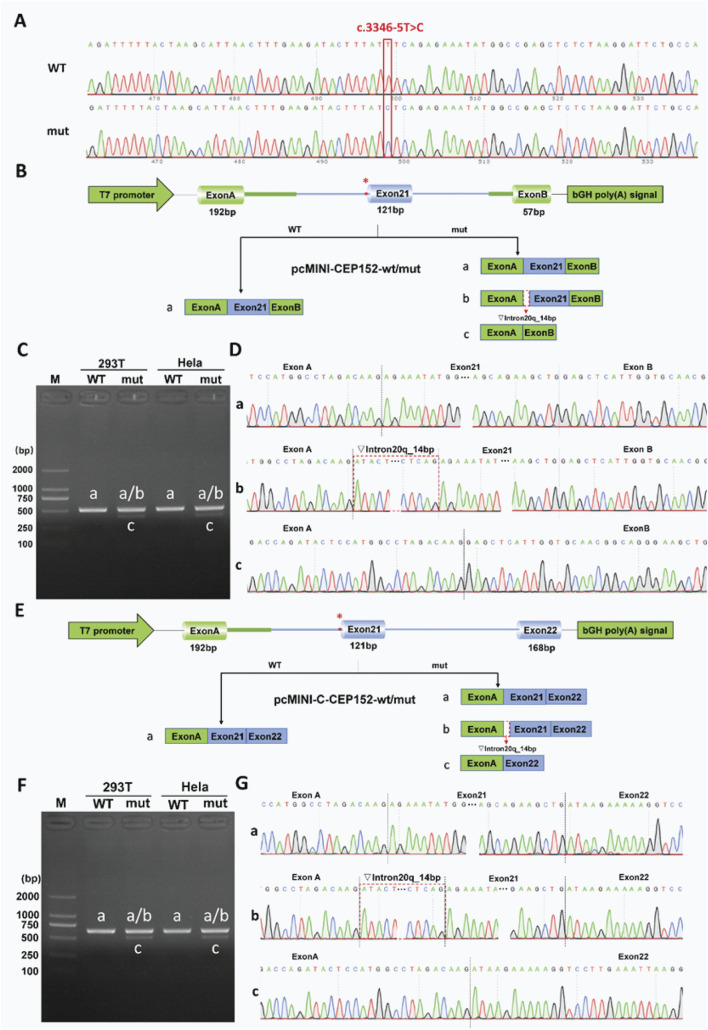
Functional analysis of the *CEP152* c.3346-5T>C variant in minigene splicing assays. **(A)** Sanger sequencing chromatograms showing wild-type (WT) and mutant (mut) alleles at the *CEP152* c.3346–5T>C locus. The red line indicates the variant position. **(B)** Schematic of *pcMINI-CEP152-wt/mut* minigene vector construction. It includes a T7 promoter, exon A (192 bp), exon 21 (121 bp), exon B (57 bp), and a bGH poly(A) signal. The asterisk marks the variant site. Three splicing patterns are shown: a for normal splicing, b for 14 bp retention of intron 20, and c for exon 21 skipping. **(C)** Agarose gel electrophoresis of RT-PCR products from 293T and HeLa cells transfected with WT and mut pcMINI-CEP152 vectors. M is the DNA marker, and bands correspond to splicing patterns in **(B)**. **(D)** Sequencing chromatograms of RT-PCR products in C, validating splicing patterns. The red dashed line shows the position of the 14 bp-retained intron 20 in pattern b. **(E)** Schematic of *pcMINI-C-CEP152-wt/mut* minigene vector construction. It contains a T7 promoter, exon A (192 bp), exon 21 (121 bp), exon 22 (168 bp), and a bGH poly(A) signal. The asterisk indicates the variant site and three splicing patterns. **(F)** Agarose gel electrophoresis of RT-PCR products from 293T and HeLa cells transfected with WT and mut pcMINI-C-*CEP152* vectors. M is the DNA marker, and bands correspond to splicing patterns in **(E)**. **(G)** Sequencing chromatograms of RT-PCR products in F, validating splicing patterns. The red dashed line shows the position of the 14 bp-retained intron 20 in pattern b.

To evaluate the pathogenic potential of the *CEP152* NM_001194998.1 c.3346–5T>C variant, initially classified as a VUS in ClinVar (accession: VCV003382300.1), we performed minigene splicing assays *in vitro* to obtain functional evidence complying with the ACMG guidelines, specifically criterion PS3 (Figure 3A). Minigene constructs were designed in two distinct configurations to ensure result reliability: the *pcMINI-CEP152-WT/mut* construct contained partial intron 20, exon 21, and partial intron 21 of *CEP152* inserted into the pcMINI vector (Figures 3B,E). *The pcMINI-C-CEP152-wt/mut* construction inserted part of intron 20, exon 21, part of intron 21, and exon 22 of *CEP152* into pcMINI-C vector (Figure 3D). Two sets of pcMINI-*CEP152*-wt/mut vectors were transfected into HeLa and 293T cells, respectively. RT-PCR results showed that the wild type produced a single band ‘a’ with a size of 510 bp in HeLa and 293T cells, while the mutant type displayed multiple bands labeled ‘a,’ ‘b,’ and ‘c.’ Single bands in the wild-type and multiple bands in the mutant in different cell lines were subjected to Sanger sequencing. (Figures 3C,F).

Sanger sequencing was further conducted to confirm the sequence of each band (Figures 3D,G). The sequencing results showed that the wild-type band ‘a’ was a normal cut band, and the cut pattern was exon A (192 bp)–exon 21 (121 bp)–exon B (57 bp). The mutant band ‘a’ was a normal cut band, and the cut pattern was exon A (192 bp)–exon 21 (121 bp)–exon B (57 bp). The mutant band ‘b’ was an abnormal splicing band, with 14 bp retention on the right side of intron 20, and the splicing pattern was exon A (192 bp)–▽intron 20 (14 bp)–exon 21 (121 bp)–exon B (57 bp). The mutant band ‘c’ was an abnormal splicing band, exon 21 was skipped, and the splicing pattern was exon A (192 bp)–Exon B (57 bp).

The consistent aberrant splicing results across the two cell lines and two vector constructs clearly demonstrate that the *CEP152* NM_001194998.1 c.3346–5T>C variant directly impairs normal mRNA splicing. Importantly, this functional evidence (ACMG PS3) is not merely a “validation” of preliminary bioinformatic predictions, which only suggested possible pathogenicity, but rather serves as a core independent criterion. When combined with clinical phenotype co-segregation, it fundamentally supports the upgrade of the c.3346–5T>C variant from VUS to pathogenic (P) in line with the ACMG guidelines.

## Discussion

MCPH–SCKS include a heterogeneous group of autosomal recessive inherited disorders characterized by primary (congenital) microcephaly and the absence of visceral abnormalities (Verloes et al., 2013; Kaindl et al., 2010; Kaindl et al., 2009; Zaqout et al., 2017). Cranial imaging demonstrates microcephaly but otherwise normal brain morphology in most cases. MCPH and SCKS were previously distinguished by patient height (the highest height in SCKS patients was only equivalent to the shortest in MCPH patients), but height is no longer a distinguishing feature because the phenotype is continuous rather than absolutely independent. The incidence of MCPH ranges from 1:30,000–1:250,000 per live birth, depending on the population (Zaqout et al., 2017). To date, the genetic etiology of MCPH is heterogeneous and comprises 28 loci, nearly all following a recessive inheritance pattern. In consanguineous families, most MCPH diagnosed cases reveal homozygous variants in the disease-causing gene. However, compound heterozygous variants are increasingly discovered in MCPH patients, raising the importance of using advanced and accurate diagnosis methods for such cases (Jean et al., 2020).

The routine prenatal ultrasound examination conducted in China for every pregnancy would identify fetuses with small head sizes, most of which would be terminated based on the abnormal ultrasound findings without genetic testing. This is probably the reason why MCPH–SCKS patients are rarely identified in China and why the *CEP152* variant spectrum and frequency have rarely been defined to date.

Although MRI can overcome shortcomings of the small field of vision and poor contrast of soft-tissue, the malformations of MCPH–SCKS patients are sometimes not limited to the head. Additionally, many patients with MCPH–SCKS are associated with genetic alterations, such as gene variants. Therefore, the use of next-generation sequencing technology for early and accurate prenatal genetic diagnosis of fetal MCPH–SCKS is of great and urgent practical significance. CNV-seq, using next-generation sequencing technology, can detect CNVs with high resolution across the whole genome. Compared with chromosome microarray technology (CMA), it can use different sequencing depths to obtain more accurate information and has the advantages of being more flexible, faster, more accurate, and lower in operation cost (PMID: 23527109). In this case, we used CNV-seq technology in the genetic diagnosis of fetal MCPH–SCKS, along with MRI and ultrasound examinations.

Hong L. L. et al. (2019) reported compound heterozygous c.1535T>A and c.3346–5T>C variants in the *CEP152* gene. In our study, we found that the proband had a heterozygous deletion of approximately 129.6 kb in the chr15:49030446–49160066 region by high-resolution CNV-seq. This deletion contained the *CEP152* and *SHC4* genes. The *CEP152* gene at chr15:49044671 with a variant of c.3346–5T>C relevant to primary microcephaly type and Seckel syndrome. The increasing use of WES has uncovered a growing number of novel and disease-causing MCPH variants (Boycott et al., 2013). WES and Sanger sequencing of the proband and the parents were further performed and showed that the variant type of the proband was hemizygous. The father carried a heterozygous variant, while the mother had no variant. The variant of the *CEP152* gene (c.3346–5T>C) in the proband was inherited from his father.

Notably, the 129.6 kb deletion encompasses *SHC4*, a gene encoding an adaptor protein involved in intracellular signal transduction (Jones et al., 2007). OMIM-cited studies show that it interacts with EGFR/MUSK and activates GRB2-mediated signaling but focus on melanocyte migration, neuro-muscular junction function, and oxidative stress responses, not neurodevelopment (Jones et al., 2007; Fagiani et al., 2007; Ahmed and Prigent, 2014). To assess whether SHC4 haploinsufficiency contributes to our phenotype, we reviewed available data. *SHC4* is not linked to microcephaly, growth retardation, or Seckel syndrome in OMIM, ClinVar, or the literature, and has no known role in MCPH–SCKS-related neuronal proliferation pathways. Although it interacts with cardiac development-related pathways, our proband had no cardiac abnormalities on prenatal imaging, and recent clinical links between *SHC4* and heart defects are irrelevant in this study (Luo et al., 2020)^.^ The proband’s severe microcephaly/growth restriction exactly matches *CEP152*-related MCPH–SCKS, with no *SHC4*-associated features (Hong L. et al., 2019; Barbelanne and Tsang, 2014). Thus, SHC4 loss does not contribute to the observed phenotype.

Our minigene splicing assays confirmed that the *CEP152* NM_001194998.1 c.3346–5T>C variant causes aberrant splicing via intron retention and exon skipping, providing critical functional evidence for its pathogenicity (ACMG criterion PS3). However, this *in vitro* model has limitations: first, the assays were performed in HeLa and 293T cell lines, which do not fully recapitulate the neuronal context, where CEP152 primarily functions in brain development; second, the minigene constructs only included partial intronic and exonic regions (intron 20 to exon 22), potentially missing long-range regulatory elements or splicing factors specific to full-length *CEP152* transcript processing. Future studies using neuronal cell models or *in vivo* systems could further validate these splicing effects in a more physiologically relevant context.

In summary, through the combined application of high-throughput sequencing technologies (CNV-seq and WES), our results showed that *CEP152* variants were the pathogenic factors that led to fetal MCPH–SCKS. Genetic testing is important for diagnosis, genetic counseling, and prenatal diagnosis. The new findings reported in this study would contribute to the investigation of the phenotype–genotype relations among patients with MCPH–SCKS.

## Data Availability

The data supporting the conclusions of this article is included within the article. CEP152 variant of c.3346-5T>C at chr15:49044671 had been submitted in ClinVar (Accession: VCV003382300.1).

## Supplementary material

SUPPLEMENTARY FIGURE S1 CNV-seq coverage depth plots for the 15q11.2 region in the family. CNV-seq coverage depth plots of chromosome 15 for the father, mother, and proband. The father (top panel) shows normal coverage across the chr15q11.2 region. The mother (middle panel) and proband (bottom panel) exhibit reduced coverage (consistent with a heterozygous deletion) in the chr15:22748952–23281864 interval, as indicated by the red line and annotated karyotype description, 46,XN,del(15q11.2).seq[GRCh37/hg19](22748952–23281864).
