# Supplementary material for: Case Report: Compound heterozygous CEP152 c.3346-5T>C variant and chr15 deletion causing recurrent MCPH–SCKS in a Chinese pregnant woman across two consecutive pregnancies
Source: Front Genet. 2025 Nov 12;16:1646297. doi: 10.3389/fgene.2025.1646297 (PMC12646542; doi:10.3389/fgene.2025.1646297)
Supplement: Supplementary file 1 [file Table1.docx]

**Table 1. List of primers used in this study**

| **Primers** | **Sequences（5′-3′）** |
| --- | --- |
| 52228-F | tcagtaagactactttctcc |
| 52436-F | gtgagctgagatcgcgccaa |
| 54180-R | atgagaaaggcagagatggt |
| 54461-R | agtctgatttttctttggcc |
| 56334-F | tccaacttcctccctctatt |
| 57465-R | cctgcttgacgtatactggg |
| pcMINI-*CEP152*-KpnI-F | ggtaGGTACCctggccttccttacaaaatt |
| *CEP152*-mut-F | ttgaagatactttatCtcagAGAAATATGGC |
| *CEP152*-mut-R | GCCATATTTCTctgaGataaagtatcttcaa |
| pcMINI-*CEP152*-XhoI-R | tttcCTCGAGaatttctagctctgaaatct |
| pcMINI-C-*CEP152*-KpnI-F | ggtaGGTACCaaagtatttagaagcatatg |
| *CEP152*-wt-F | cttgtattcagagctactggctcctatgtc |
| *CEP152*-wt-R | gacataggagccagtagctctgaatacaag |
| pcMINI-C-*CEP152*-XhoI-R | TAGACTCGAGCTCCAATTTTTTCCACTACA |
| pcMINI-F | CTAGAGAACCCACTGCTTAC |
| pcMINI-R | TAGAAGGCACAGTCGAGG |
| pcMINI-C-F | CTAGAGAACCCACTGCTTAC |
| pcMINI-C-R | TAGAAGGCACAGTCGAGG |
