# Supplementary figures and images for: Case Report: Compound heterozygous CEP152 c.3346-5T>C variant and chr15 deletion causing recurrent MCPH–SCKS in a Chinese pregnant woman across two consecutive pregnancies
Source: Front Genet. 2025 Nov 12;16:1646297. doi: 10.3389/fgene.2025.1646297 (PMC12646542; doi:10.3389/fgene.2025.1646297)

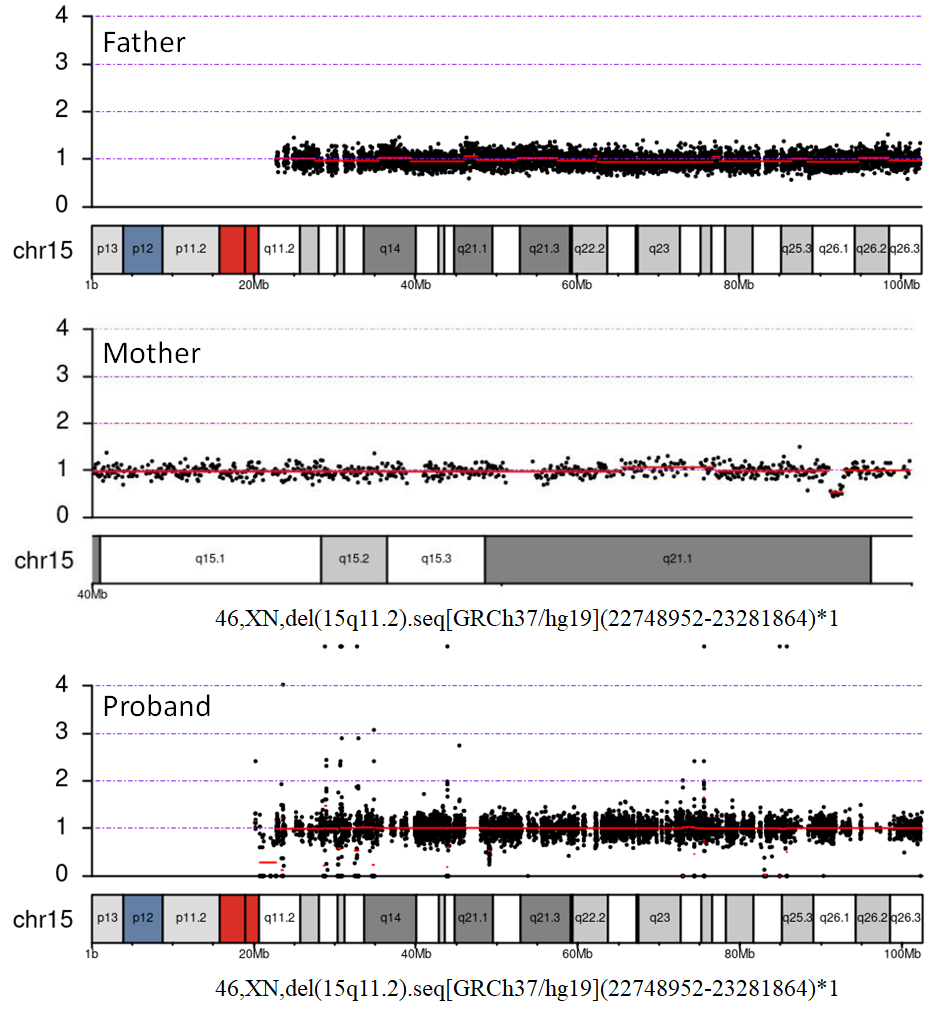

Supplement: Supplementary file 2 [file Image1.tif]
